# The Role of Concentration and Solvent Character in the Molecular Organization of Humic Acids

**DOI:** 10.3390/molecules21111410

**Published:** 2016-10-27

**Authors:** Martina Klučáková, Kateřina Věžníková

**Affiliations:** Materials Research Centre, Faculty of Chemistry, Brno University of Technology, Purkyňova 118/464, Brno 612 00, Czech Republic; xcveznikova@fch.vutbr.cz

**Keywords:** humic acid, molecular organization, conformation, aggregation, ultrasound spectrometry

## Abstract

The molecular organization of humic acids in different aqueous solutions was studied over a wide concentration range (0.01–10 g·dm^−3^). Solutions of humic acids were prepared in three different media: NaOH, NaCl, and NaOH neutralized by HCl after dissolution of the humic sample. Potentiometry, conductometry, densitometry, and high resolution ultrasound spectrometry were used in order to investigate conformational changes in the humic systems. The molecular organization of humic acids in the studied systems could be divided into three concentration ranges. The rearrangements were observed at concentrations of ~0.02 g·dm^−3^ and ~1 g·dm^−3^. The first “switch-over point” was connected with changes in the hydration shells of humic particles resulting in changes in their elasticity. The compressibility of water in the hydration shells is less than the compressibility of bulk water. The transfer of hydration water into bulk water increased the total compressibility of the solution, reducing the ultrasonic velocity. The aggregation of humic particles and the formation of rigid structures in systems with concentrations higher than 1 g·dm^−3^ was detected.

## 1. Introduction

The molecular organization of humic acids in aqueous solutions and their conformational changes play a significant role in their behaviour and functioning in Nature. Due to their heterogeneous and polydisperse character, their secondary structure and conformational arrangements has not been clarified yet [1,2,3]. The significance of the secondary structure of dissolved humic substances in their interactions with other compounds present in natural systems has been attributed to entrapment in structural voids, adsorption on surfaces, and partitioning into the hydrophobic interiors of micelle-like structures and humic aggregates [3,4,5,6]. It is well known that diverse humic particles progressively aggregate in response to solution parameters such as pH, ionic strength, the presence of multiply charged cations, temperature, and concentration [3,7,8]. De Moreas and Rezende [9] showed that the formation of micelles depends on the number and nature of hydrophobic association sites in humic solutions and on the origin and concentration of humic acids at a given pH value. At lower pH values, the possibility of humic molecules forming pseudomicelles increases due to a combination of neutralized and dissociated charged sites. At high ionic concentrations, organizational rearrangements result in a salting-out effect, i.e., the formation of a hydrophobic colloid. At lower salt concentrations, the formation of pseudo-micelles precedes intermolecular interactions [10]. Longstaffe et al. [11] studied the relationships between pH and the distribution of binding interactions at different domains of dissolved humic acids for xenobiotics. They showed that the pH-dependence of the interactions can be correlated with changes in the conformation of the carbohydrate components of humic acids rather than with the aromatic components. It was argued that the observed preference for aromatic humic structures results from restricted access to the non-aromatic humic components at low pH values. These humic components form tightly bound hydrophobic domains due to strong inter- and intra-molecular hydrogen bonds. At high pH values, these structures can open up, making them more available for interactions with polar compounds. Alvarez-Puebla et al. [12] modelled fulvic acid as a function of its ionic state under different conditions. They stated that the presence of water molecules had a stabilization effect on the electrostatic energy, which was greater as ionized rate increases and the nonionized aggregated species were more stable than monomers because of the increase in their interaction due to H-bonding and non-bonding forces. No aggregation process was observed for the ionized molecules. Critical factors for the aggregation were the concentration and the ionic state. Colombo et al. [13] used atomic force microscopy to investigate the molecular dynamics of humic acid aggregate formed at different pH values. Their observations suggested that humic acids under pH 5 exhibited a pseudo-amphiphilic nature, with secluded hydrophobic domains and polar subunits in direct contact with hydrophilic surface. They carried out the molecular simulation in order to explain the humic ring-like morphology which resulted in an optimized structure comprising 45–50 linear helical molecules looped spirally around a central cavity. In the absence of hydration forces and at acidic pH, the conformation was stabilized by intermolecular dipole-dipole and van der Waals interactions.

The environmental functioning of humic substances is often controlled by the content of acidic functional groups and their positions in the conformational structures [12,14]. The particle size and molecular conformation depend on the concentration of humic particles and counter-ions. The reactions of acidic groups can therefore affect the molecular sizes of humic particles and the stability of the molecular conformation [15]. Arilstide and Sposito [16] studied molecular dynamics in interactions of ciprofloxacin either with protonated humic substances or deprotonated ones bearing Ca(II), Mg(II), and Fe(II) ions. They stated that humic substances underwent conformational changes through rearrangements of their hydrophobic and hydrophilic regions and the disruption of their intramolecular H-bonds to facilitate favourable intermolecular H-bonding interactions. Jung et al. [1] studied the local molecular organization of natural and synthetic humic acids by means of fluorescence and surface tension measurements. Their results showed that hydrophobic interactions are responsible for the organization of the aggregates, regardless of the origin of humic acids. The specific interactions of aromatic molecular sites depend on the whole tri-dimensional environment of humic acids in solution. Surface tension measurements revealed an increase in surface activity as a function of concentration for humic acids and their fractions.

In this work, the molecular organization and conformational changes of humic acids were studied by means of an innovative method: high resolution ultrasound spectrometry. It is a modern technique for the direct and non-destructive analysis of the intrinsic properties of materials and is based on measurements of the velocity and attenuation of an ultrasonic wave propagating through a sample. The ultrasonic velocity is determined by the density of the sample and its elastic response to the oscillating pressure (stress) of the ultrasonic wave, and can be expressed in terms of compressibility or the (longitudinal) storage modulus. The elastic response is generally dominant. The attenuation, which is a measure of the ultrasonic transparency of the medium analysed, is determined by the energy losses in the compressions and decompressions in the ultrasonic waves and so can be expressed in terms of the viscosity of the medium or its longitudinal loss modulus [17,18,19]. This parameter is extremely sensitive to the molecular organization and intermolecular interactions in the analysed medium, which, in the case of humic substances and their complexes, can strongly influence their reactivity and structural arrangement [20,21,22,23]. The ultrasonic analysis is based on the measurement of characteristics of ultrasonic waves propagating through a sample. In general, sound can be absorbed by matter, which either attenuates the signal or changes its velocity. Ultrasonic attenuation (N) describes the decay of the amplitude of an ultrasonic wave with distance travelled. The ultrasonic velocity (U) is the speed of this wave and is related to the wavelength and the frequency of oscillation of the deformation. It is determined by the density and compressibility of the sample [24]:

U = 1/√βρ,
(1)

where U is the ultrasound velocity of the sample in cell 1, β is the compressibility, and ρ is the density of the sample. The computed compressibility is usually normalized to the compressibility of water (β_w_):

β_r,1_ = β/β_w_, (2)

or to the compressibility of the surrounding medium (β_m_):

β_r,2_ = β/β_m_, (3)

The compressibility is determined by the elastic response of the sample to the oscillating pressure (stress) in the ultrasonic wave. This parameter is extremely sensitive to the molecular organization and intermolecular interactions in the analysed medium [2,25,26].

## 2. Results and Discussion

**Figure 1:**
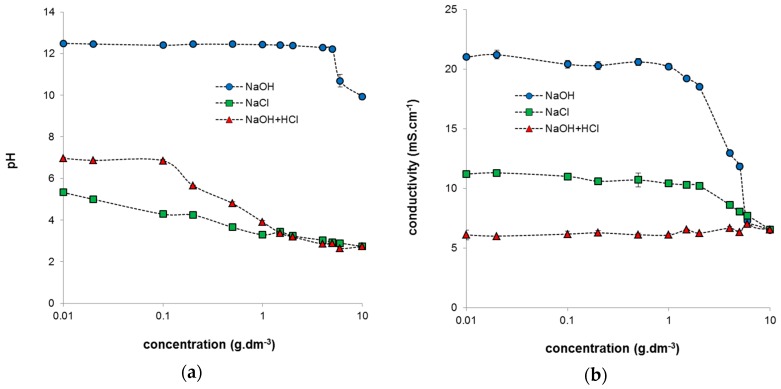
Obtained pH values (**a**) and conductivity values (**b**) of prepared humic sols in dependence on concentration of humic acids.

Figure 1 shows the changes in pH and conductivity for the studied systems. We can see that the systems differ in both measured quantities and their values depend on the concentration. In the case of humic acids dissolved in NaOH, the pH values are practically constant over a wide concentration range and decrease slightly only for highly concentrated solutions. Similarly, a decrease in conductivity was observed only for very concentrated systems.

**Figure 2:**
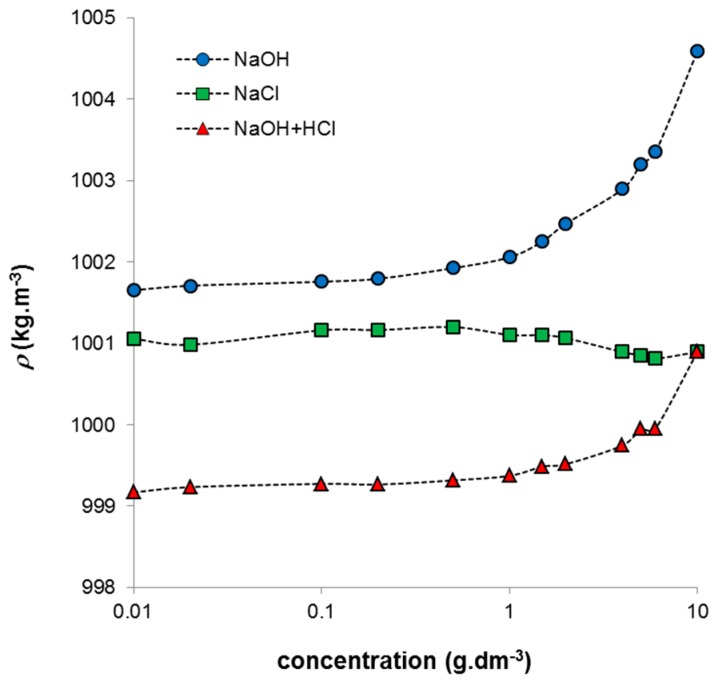
Obtained density of prepared humic sols in dependence on concentration of humic acids.

The results obtained for systems containing NaCl differ significantly from those for alkaline humic solutions. If the stock solution (10 g·dm^−3^) was diluted by NaCl solution, lower pH values were measured and they decreased with increasing humic concentration. The conductivity decreased only for very concentrated systems similarly as in the case of humic acids in NaOH. The systems prepared by neutralization of the alkaline humic solution by HCl exhibited practically constant conductivity at all used concentrations and their pH values decreased with increasing concentration. Since the ionic strength of all the prepared systems was the same, the described changes were given only by changes in humic concentration in combination with the nature of the solvent medium. The density of the studied systems increased strongly mainly at higher concentrations with the exception of the systems diluted by NaCl, which have practically constant density in whole concentration range (Figure 2). The increase can be caused by higher degree of intermolecular interactions in more concentrated systems. Changes in density resulted also in the measured ultrasonic velocity which is given by the density and compressibility of studied system (Equation (1)). In the case of the increase in density the ultrasonic velocity decreases. The increase of the compressibility has similar effect.

Some authors propose that dissolved humic acids might be micelle-like, supra-molecular assemblies of small entities. The apparent conformation of humic acids at higher concentrations (usually >1 g·dm^−3^) might differ significantly from the conformation in more dilute systems [2,12,27,28,29,30,31].

Our results from measurements of conductivity, pH, and density correspond with this finding. A critical examination of published data suggests that humic substances are collections of diverse, relatively low molecular mass components forming dynamic associations stabilized by hydrophobic interactions and hydrogen bonds. These associations are capable of organizing into micellar structures in suitable aqueous environments [2,29,30,31,32].

The systems studied in this work differ in their composition and in their method of preparation. Humic systems of first two types can be defined as phase colloids, i.e., humic particles of colloidal size dispersed in the NaCl solution and stabilized by electric double layer. The preparation procedures of the systems were different. In the first case, NaCl was prepared by means of the neutralization of NaOH by HCl, in the second case, the NaCl solution was used in the dilution of the stock solution. Third type of studied system is the solution of humic acids in NaOH. This type differed from others mainly by the composition. We can see that the neutralization of alkaline humic solutions at every used concentration led to different results, as in the case of simple dilution by NaCl. It seems that the “fresh” dissolution in NaOH and subsequent neutralization facilitated the dynamic development of a secondary structure of humic particles in these systems. In the case of simple dilution by NaCl, the system had only limited possibilities of re-arrangement, which were manifested in the constant density of the system and a gradual increase in pH with increasing dilution.

**Figure 3:**
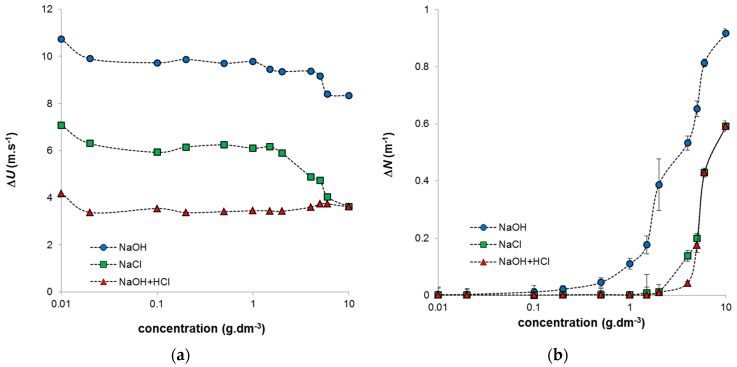
Obtained ultrasound parameters—velocity ΔU (**a**) and attenuation ΔN (**b**) of prepared humic sols in dependence on concentration of humic acids.

The ultrasonic measurements provided data characterizing the organization of humic particles in studied systems. As we can see the main changes in obtained ultrasonic parameters (velocity and attenuation), the main changes were observed for highly concentrated systems. Intramolecular interactions resulted in the increase of particle size. The ultrasonic velocity decreased also in region of low concentrations where the density of studied systems was practically constant. In this case, the compressibility had the majority influence on the speed of ultrasonic wave in sample. Obtained values of ultrasonic parameters thus charicterized the hydration shells in the used humic sols and their elasticity. In measurements of ultrasound velocity, the elasticity is usually dominant [2,20], which was evident mainly for the constant region of density values. It can be seen that, as expected, the ultrasound velocity was higher in the humic solution than in the water in the reference cell. We can observe several breaks in the concentration dependence of ΔU. The first one was observed in the region of low concentrations (~0.02 g·dm^−3^) for all studied systems. The decrease in ultrasonic velocity was probably caused by the release of hydration water from the coordination shell of the humic acids. The compressibility of water in the hydration shells is less than the compressibility of bulk water; therefore, the transfer of hydration water into the bulk water increases the total compressibility of the solution, reducing the ultrasonic velocity. This low-concentration region was evaluated by Jung et al. [1] as the region where molecular weak-adsorption interactions between the pyrene fluoroprobe and the most hydrophobic sites of humic acids take place. In accordance with this, our results indicate that intra- and inter-hydrophobic interactions can take place in humic systems at low concentrations. Only one break was observed for systems prepared by neutralization of the alkaline humic solution by HCl, and the ΔU values were practically constant for the whole concentration range above 0.02 g·dm^−3^ (Figure 3a). In contrast, systems prepared by dilution using NaCl and alkaline humic systems in NaOH had a constant region between 0.02 and 1 g·dm^−3^, after which their ΔU values decreased with increasing humic concentration. The decrease was stronger for humic/NaCl systems, probably as a result of the different stabilities of their humic secondary structures.

It is well known that humic acids can be relatively easily dissolved in alkaline solutions including NaOH. These systems can be regarded as true solutions with humic particles dissolved in the surrounding medium. The systems are the true solution within the meaning of a homogeneous mixture of two components. One of them (humic acids) is dissolved in other one (NaOH) and no phase interface can exist between solute and solution. In the case of humic acids, the dissolved particles can be bigger than 1 nm which is considered as the upper limited value for analytical solutions, therefore the use of this phrase should express the absence of phase boundary (not the size of dissolved particles). Due to the high pH values of these systems, their acidic functional groups are mostly dissociated. The increase in humic content in these systems can thus result in a smaller space for the unrolling of humic chains and therefore the observed changes in the measured ultrasound parameters. By contrast, systems prepared by the neutralization of humic acids in NaOH using HCl can be considered as phase (irreversible) colloids stabilized by their surface charges. The interface between humic particles and the surrounding medium allows ions of opposite charge to be adsorbed and influences their coagulation (or aggregation) behaviour. The functional groups of humic acids are to some degree protonated as a consequence of lower pH values. The subsequent dilution by NaCl can influence only the ratio between the content of humic particles and the volume of the surrounding medium and not its ionic strength. Nevertheless, the ratio between the amount of humic particles present and the amount of counter-ions changes with the dilution, which results in changes in the measured ΔU values. The re-arrangement of the secondary structure in more concentrated humic systems usually includes the formation of micellar species or aggregates [1,7,8]. The formation of larger species can be confirmed by the measured attenuation in the studied humic systems (Figure 3b). The increase in the obtained ΔN values, observed mainly in the case of the systems with evident changes in ΔU values at higher concentrations, confirmed the increase in size of humic species and their partial sedimentation. The attenuation characterized the ultrasonic transparency of the sample and can be seen as a reduction in the amplitude of the wave. Its increase is usually related to the aggregation of humic acids in more concentrated systems, a fact which has been observed by means of ultrasound spectrometry or other analytical methods [2,17,20,27,28,29,30,31]. In our study, the ΔN values were higher for alkaline humic solutions, which had better possibilities to form a stable secondary structure. Humic acids in alkaline systems are able to build more rigid structures, these corresponding to strong increases in attenuation in highly concentrated alkaline humic systems.

**Figure 4:**
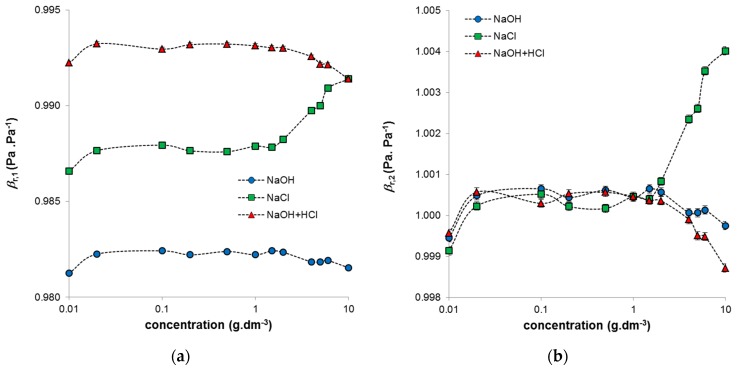
Obtained compressibility of prepared humic sols normalized on water (**a**) and the surrounding medium (**b**) in dependence on concentration of humic acids.

Ultrasound velocity and density were used to determine the compressibility of the studied humic systems, this being the parameter sensitive to their conformational changes (Equation (1)). The elastic response is extremely sensitive to molecular organization and intermolecular interactions. As mentioned previously, the conformation in higher concentrations of humic acids (∼1 g·dm^−3^) may differ significantly from the conformation in more dilute systems. Conformational changes in dissolved humic acids at low concentrations (<1 g·dm^−3^) have been demonstrated by many authors [27,29]. In our study, the determined values of compressibility were normalized to the compressibility of water (Equation (2)) or to the compressibility of the surrounding medium (Equation (3)). Both types of normalized compressibility showed that their concentration dependencies can be divided into three regions (Figure 4). The first is a region of very low humic concentrations. For all studied systems, a break was observed at around a concentration of 0.02 g·dm^−3^. This break was caused by changes in elasticity, as the densities of the systems were practically constant in this region (see Figure 2); similar breaks were observed in the measured ΔU values (see Figure 3a). The region of constant compressibility between 0.02 and 1 g·dm^−3^ corresponds with the measured values of both density and ultrasound velocity. In this region, the highest affinity of humic acid to copper was observed. The organization of more concentrated solutions changes, and the humic particles can aggregate, which results in worse accessibility to the active sites and a decrease in the amount of bonded copper [25]. The difference in the compressibility of humic acids in NaOH and NaOH neutralized by HCl is in the normalization. If the compressibility was normalized to water (Figure 4a), the values for these two systems differed, but if they were normalized to the given medium (Figure 4b), their values were practically the same. In contrast, systems diluted by NaCl behaved differently. The compressibility of highly concentrated systems (>1 g·dm^−3^) increased significantly. This increase was clearly caused by the decrease in ultrasound velocity, as the densities of these systems were practically constant over the whole used concentration range. The increase in compressibility is probably connected with changes in the hydration shell of humic acids leading to its greater elasticity. In this study, the stock solution (10 g·dm^−3^), prepared by dissolving humic acids in NaOH and by adding the same amount of HCl, was gradually diluted by NaCl. Therefore these systems were prepared from the highest concentration. The dilution changed the ratio between humic acids and NaCl, which resulted in the transfer of hydration water into the coordination shell of the humic acids and a strong increase in the compressibility of these systems.

## 3. Materials and Methods

Humic acids were extracted from South-Moravian lignite using a mixture of NaOH and Na_4_P_2_O_7_ by the procedure described and characterized previously [19,33,34,35,36,37]. The basic characteristics of the extracted humic sample are listed in Table 1. It was characterized according to elemental composition using a Flash CHNSO Microanalyzer 1112 (Thermo Scientific, Delft, The Netherlands). The total acidity and content of carboxylic groups were determined by standard methods [21,34,37].

Three different types of solutions were used in this work. The first type was a colloidal solution prepared by the dissolution of humic acids in 0.1 M NaOH and the addition of the same volume of 0.1 M HCl. It means that humic acids were mixed with the NaOH solution and stirred up to their complete dissolution. Then, the HCl solution was added. This procedure resulted in the neutralization of NaOH by HCl and humic acids should be surrounded by the NaCl solution (formed directly in the preparation of this system). Because of neutralization, the surrounding medium should have had a neutral pH-value, but the presence of humic acids caused an increase in the acidity of the system in dependence on their concentration. All systems of first type (different concentrations) were prepared by the above mentioned procedure, i.e., every concentration of humic acids was the result of their dissolution in NaOH and the neutralization by HCl. The second type was a solution prepared by diluting the stock solution of the previous type with 0.1 M NaCl. The concentrated solution (10 g·dm^−3^) prepared by the above described procedure (the dissolution of humic acids in NaOH followed by the addition of HCl) was diluted with 0.1M NaCl. The NaCl solution used for the dilution was prepared by the dissolution of NaCl in water. Solutions of both these types have the same chemical character but are prepared by different procedures, which can influence the final organization of humic particles. The third type was an alkaline solution in 0.1 M NaOH. The concentration range of humic acids in their solutions was 0.01–10 g·dm^−3^. The prepared humic sols were characterized by means of pH and conductivity (both using a Five Easy Plus instrument (Mettler Toledo, Prague, Czech Republic).

An HR-US 102 high resolution ultrasonic spectrometer (Ultrasonic Scientific, Dublin, Ireland) was used to measure basic ultrasonic parameters at three frequencies (5110 kHz, 8220 kHz, and 12 200 kHz). Six values for each ultrasonic parameter were measured at each frequency and averaged. The device consisted of two independent cells heated to 25 °C. Cell 1 was used for the humic solution, cell 2 for water. The ultrasonic velocity and attenuation in both cells were monitored; the resulting values ΔU and ΔN were calculated as the differences between the values determined for the sample and reference cells. The densities of humic sols were determined by a DMA 4500 (Anton Paar, Graz, Austria).

## 4. Conclusions

In this work, the molecular organization of humic acids in three different surrounding mediums was studied. It was found that the conformational arrangement changed with the concentration of humic acids and the nature of their environment. In general, the organization of humic systems can be divided into three parts. The first is a region of very low concentrations (<0.02 g·dm^−3^). The compressibility of water in the hydration shells is less than the compressibility of bulk water. Therefore, the transfer of hydration water into the bulk water increases the total compressibility of the solution, reducing the ultrasonic velocity. The rearrangement of humic particles at these low concentrations is connected with changes in their hydration shells and elasticity. In our study, the high resolution of the ultrasound spectrometer and the very precise measurement of density allowed these changes to be detected on the basis of the determined ultrasound parameters. No significant changes in the studied parameters were observed in the concentration range between 0.02 and 1 g·dm^−3^. Probably, a relatively stable secondary structure for the humic acids was established; in addition, a “switch-over point” in the molecular organization of the humic acids was detected at 1 g·dm^−3^. The aggregation and formation of a rigid structure was observed above this concentration. It was observed that systems prepared by dilution of the stock solution with NaCl exhibited a different behaviour at high concentrations in comparison with the other studied systems. The increase in compressibility and decrease in ultrasound velocity (with only a slight variation in the density) indicated the significant transfer of water from the hydration shells. They behaved as phase (irreversible) colloids stabilized by their surface charges, which influence significantly their conformational changes. In the case of simple dilution of stock solution by NaCl, the system had only limited possibilities of re-arrangement, which were manifested in the constant density of the system, strong decrease of ultrasound velocity, and increase in the compressibility of this system.

## Figures and Tables

**Table 1:** Elemental composition, total acidity, and carboxylic acidity of humic acids.

C (at. %)	H (at. %)	N (at. %)	S (at. %)	O (at. %)	Total Acidity (mmol·g^−1^)	Carboxylic Acidity (mmol·g^−1^)
42.3	40.2	1.3	0.1	16.1	4.58	2.74
